# Cardiovascular involvement in Epstein–Barr virus infection

**DOI:** 10.3389/fimmu.2023.1188330

**Published:** 2023-05-22

**Authors:** Xinying Chen, Yingying Li, Lijun Deng, Lianyu Wang, Wenting Zhong, Junbin Hong, Liyu Chen, Jinghua Yang, Bin Huang, Xiaolan Xiao

**Affiliations:** ^1^ Department of Pediatrics, The Second Affiliated Hospital of Guangzhou University of Chinese Medicine, Guangzhou, China; ^2^ The Second Clinical Medical College, Guangzhou University of Chinese Medicine, Guangzhou, China; ^3^ Ying Lv’s Expert Inheritance Studio, Guangdong Provincial Hospital of Chinese Medicine, Guangzhou, China

**Keywords:** cardiovascular damage, coronary artery lesions (CALs), myocarditis, Epstein–Barr virus (EBV), immune injury

## Abstract

Cardiovascular involvement is an uncommon but severe complication of Epstein–Barr virus (EBV) infection caused by direct damage and immune injury. Recently, it has drawn increasing attention due to its dismal prognosis. It can manifest in various ways, including coronary artery dilation (CAD), coronary artery aneurysm (CAA), myocarditis, arrhythmias, and heart failure, among others. If not treated promptly, cardiovascular damage can progress over time and even lead to death, which poses a challenge to clinicians. Early diagnosis and treatment can improve the prognosis and reduce mortality. However, there is a lack of reliable large-scale data and evidence-based guidance for the management of cardiovascular damage. Consequently, in this review, we attempt to synthesize the present knowledge of cardiovascular damage associated with EBV and to provide an overview of the pathogenesis, classification, treatment, and prognosis, which may enhance the recognition of cardiovascular complications related to EBV and may be valuable to their clinical management.

## 1 Introduction

Epstein–Barr virus (EBV) is prevalent in people worldwide and can even last a lifetime. In contrast to EBV infections in most children, which are typically asymptomatic or have ambiguous symptoms, infections in adolescents and adults frequently result in infectious mononucleosis (IM), a self-limiting disease with a favorable prognosis (1, 2). However, for those with an immune deficiency or impairment, EBV could lead to aggressive and even catastrophic diseases, such as chronic active EBV (CAEBV) disease, hemophagocytic lymphohistiocytosis (HLH), and specific tumors, among others (3, 4). It has been hypothesized that immune escape, infected T or natural killer (NK) cell clonal expansion, and invasion into systemic organs, which causes their failure, are related to EBV infection (4). EBV can affect not only the lymphocytes but also other systems, and its prognosis depends on the manifestations and the severity of complications (1–5).

Cardiovascular involvement in EBV infection is a type of severe complication, which includes coronary artery dilatation (CAD), coronary artery aneurysm (CAA), myocarditis, valvular heart disease, heart failure, and pulmonary arterial hypertension (PAH), among others, that can be fatal if left untreated (6–9). As reported previously, the cardiovascular injuries associated with active EBV (AEBV) are primarily myocarditis, pericardial disease, and heart failure, while cardiovascular damage in patients with CAEBV manifests as coronary aneurysms or myocarditis (6, 8). In a nationwide survey in Japan, patients with CAEBV presented with myocarditis in 6% of cases and CAA in 9% (5). According to two other studies, approximately 9.4%–17.9% of patients with CAEBV experience circulatory system complications (10, 11). However, there is a lack of reliable large-scale data and evidence-based guidance for the treatment of cardiovascular damage, which is challenging for clinicians. This review aimed to summarize the current progress on the pathogenesis, diagnosis, treatment, and prognosis of the different cardiovascular complications associated with EBV. A literature review is shown in **Table 1**.

**Table 1:** Literature review of the cardiovascular involvement in Epstein–Barr virus infection.

First author (year/country)	Age/gender	Diagnosis	Cardiovascular complications	Prognosis
Chimenti (2002/Italy) (6)	67 years/F	AEBV	Infarct-like myocarditis; coronary vasculitis; pericardial disease (mild pericardial effusion); multiple left ventricular (LV) microaneurysms; moderate LV dysfunction	Functioning of the left ventricle improved.
Jamal (2021/Morocco) (12)	22 years/M	CAEBV; HLH	Giant CAA	He died 10 days after the start of the conditioning regimen, following a second cerebral aneurysm rupture.
Hastie (2021/Australia) (13)	57 years/F	AEBV	Pericardial effusion causing subacute cardiac tamponade	She was in stable condition.
Aknouk (2022/America) (14)	20 years/M	AEBV	Myocarditis; dilated cardiomyopathy; acute congestive heart failure; multivalvular regurgitation	The patient eventually died.
Ho (2015/England) (15)	80 years/F	AEBV	Pericardial effusion causing subacute cardiac tamponade	The pericardial effusion disappeared after 2 months.
Kang (2020/Japan) (16)	42 years/M	CAEBV	CAD; CAA	CAD and CAA both progressed.
Watanabe (2019/Italy) (17)	20 years/F	AEBV	Myocarditis; pericardial effusion; heart failure; malignant ventricular arrhythmias and cardiac arrest	The patient was kept on medication
Paul (2019/America) (18)	63 years/M	AEBV	Myocarditis	He died 1 week later.
Pi (2022/China) (19)	36 years/F	CAEBV	Massive pericardial effusion	Pericardial effusion disappeared
Takano (2008/Japan) (20)	45 years/M	CAEBV	Myocarditis; mild pericardiac effusion; heart failure	He died of multiple organ failure and disseminated intravascular coagulation.
Ba (2019/China) (7)	9 years/M	CAEBV	CAD; CAA; PAH	He was kept on medication: prednisone.
Li (2022/China) (21)	5 years/F	CAEBV	CAA; giant sinus of valsalva aneurysm (SOVA); heart valve disease	She died of an undetermined cause without receiving any further care.
Kawamura (2016/Japan) (9)	4 years/F	EBV-HLH	Acute myocarditis; sudden cardiopulmonary arrest; giant CAA	No sequelae except for a CAA
Jiang (2016/China) (22)	16 years/F	CAEBV	Aortic aneurysm; CAA; coronary arteries stenoses; myocardial infarction	Coronary artery occlusion
Xiao (2020/China) (8)	4 years/F	CAEBV	CAA; heart valve disease (mitral and aortic valve insufficiency)	Her CAAs did not progress.
Chen (2020/China) (23)	17 months/M	EBV-HLH (HLH-2004); XLP (the variant c.116G > C);	CAD; heart valve disease (valvular regurgitation); pericardial disease (pericardial effusion)	He was in stable condition.
Jin (2017/China) (24)	1 year, 10 months/F	CAEBV; EBV-HLH (HLH-2004)	CAD; pericardial disease (pericardial effusion)	Her coronary artery dilatation progressed after 2 weeks, and she discontinued treatment for financial reasons. After 4 months, she died.
Liu (2020/China) (25)	1 year, 5 months/M	AEBV; XLP	CAD	His CAD returned to normal 42 days after admission.
Shi (2015/China) (26)	20 months/F	Reactivated EBV	CAD	Her coronary artery recovered after 1 week.
Liu (2017/China) (27)	(3.47 ± 1.61 years)/M (8) and F (7)	AEBV (IM)	CAD	All 13 cases of coronary dilatation returned to normal. Two cases did not return to normal and are still being followed up.
Xie (2016/China) (28)	3 years/M	CAEBV	CAA	They were all kept on medication.
	2 years/M	CAEBV	CAD, CAA	
	6 years/F	CAEBV; HLH	CAA	
	4 years/F	CAEBV	CAD; CAA; heart valve disease (moderate mitral regurgitation)	
Teng (2022/China) (29)	9 years/F	CAEBV	CAD; heart valve disease (mitral regurgitation)	The patient’s symptoms improved, but the CAD did not return to normal. Her condition was stable.
Shao (2016/China) (30)	1 year, 5 months/M	CAEBV	Right CAA; left CAD	The family refused antiviral therapy and left.
Wei (2021/China) (31)	6.05 (2.8–14.3 years)/M (6 cases) and F (4 cases)	CAEBV (10 cases); complicated with HLH (3 cases)	Bilateral CAD (8 cases) + unilateral CAD (2 cases); mild mitral and aortic valve regurgitation (3 cases); mild tricuspid regurgitation (3 cases); pericardial effusion (4 cases)	The CAD of 3 patients had returned to normal.
Sui (2018/China) (32)	17 years/M	AEBV	Myocardial calcification; myocarditis; heart failure	He was alive.
McCrory (2018/America) (33)	10 years/M	EBV-HLH	Cardiac arrest	The family elected to discontinue ventilator support after brain death.
Ba (2019/China) (34)	10 years/M	CAEBV	Bilateral CAD and thrombosis; aortic dilatation; bilateral pulmonary artery dilatation; PAH; abdominal aortic dilatation and distal superior mesenteric artery dilatation	One year later, he developed generalized edema, shortness of breath, and decreased activity tolerance. The LAD and pulmonary artery further expanded, and a moderate amount of pericardial effusion appeared. Subsequently, antiviral, pulmonary artery pressure-lowering, diuretic, and other treatments were added. The shortness of breath disappeared and the activity tolerance improved.
Sun (2021/China) (35)	10 years/F	EBV-HLH	CAAs; pericardial effusion	Pericardial effusion disappeared, but CAAs remained.

F, female; M, male; AEBV, active Epstein–Barr virus; CAEBV, chronic active Epstein–Barr virus; HLH, hemophagocytic lymphohistiocytosis; XLP, X-linked lymphoproliferative disease; CAD, coronary artery dilation; CAA, coronary artery aneurysm; PAH, pulmonary arterial hypertension; LAD, left anterior descending artery; IM, infectious mononucleosis.

## 2 Pathogenesis

**Figure 1:**
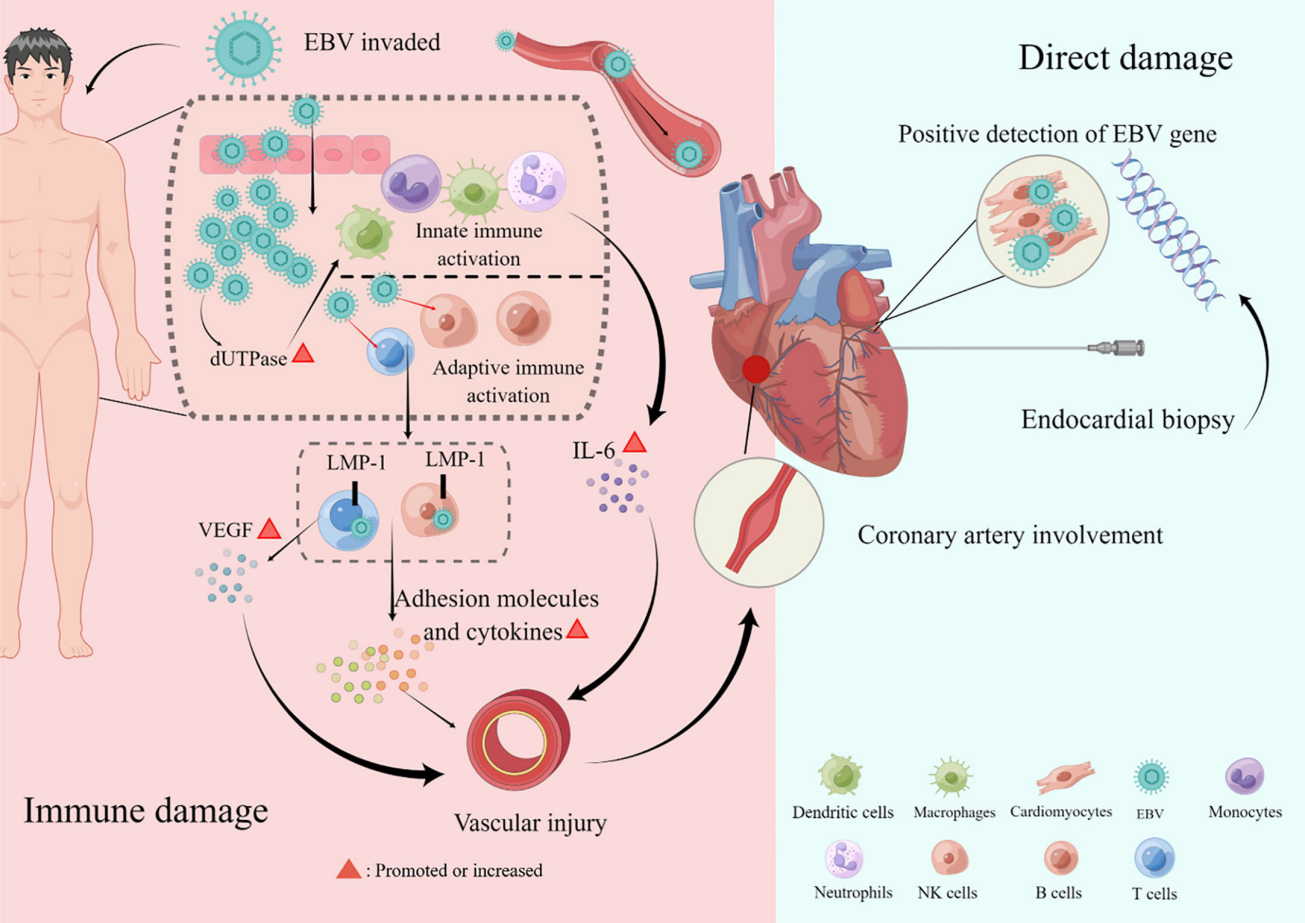
Pathogenesis of cardiovascular damage. The mechanism of cardiovascular damage caused by Epstein–Barr virus (EBV) infection is mainly divided into direct and indirect damage. Direct damage refers to the positive detection rate of the EBV virus gene in endomyocardial samples. Indirect damage, also called immune damage, consists of innate and adaptive immune activations that cause cardiac involvements, such as coronary artery lesions, myocarditis, pericardial diseases, valvular heart disease, and heart failure. This figure was drawn on the Figdraw website (https://www.figdraw.com/static/index.html).

The mechanism of cardiovascular damage caused by EBV infection is not fully understood, but it is mainly classified into direct and indirect damage (**Figure 1**). Evidence of direct EBV damage comes from viral genetic testing of endomyocardial samples from patients with cardiomyopathy, which is also the gold standard. In several studies, the positive detection rate of the EBV virus gene in endomyocardial samples was 2%–4% (36, 37). The exact pathogenesis of myocarditis caused by EBV is limited due to the lack of adequate animal models. However, in autopsies of patients with EBV-associated myocarditis, it was revealed to be accompanied by a severe inflammatory infiltration, with the absence of direct invasion in cardiomyocytes under the electron microscope, suggesting that it was not caused by direct damage (38). Furthermore, in an animal model, B- and T-lymphocyte-deficient mice infected with herpesviruses did not develop myocardial necrosis despite high viral loads, demonstrating that viral replication cannot fully explain the myocardial injury. Currently, it is more often proven that EBV-related cardiovascular involvement is an indirect injury, also known as immune injury, and immune cells mediate cardiovascular damage. As for coronary artery involvement (CAI), pathological studies have confirmed that EBV-associated coronary injury is caused by lymphoid vasculitis (39). There is no doubt that EBV-associated vasculitis involves local inflammatory cell chemotaxis, recruitment, adhesion, infiltration, cytotoxic injury, and cytokine secretion (40). On the one hand, latent membrane protein 1 (LMP-1), a surface EBV antigen marker of cytotoxic T cells (CTLs) infected with EBV, can enhance the production of vascular endothelial growth factor (VEGF), and VEGF can enhance the post-capillary permeability of veins and venules, leading to vascular involvement and the degradation of vessel walls (41). On the other hand, EBV-positive natural killer (NK)/T cells promote the secretion of adhesion molecules and cytokines, aggravating vascular lesions (42). In addition, EBV will produce deoxyuridine triphosphatase (dUTPase) during the replication process, which will increase the level of interleukin 6 (IL-6), cause vascular endothelial damage, and lead to coronary artery abnormalities (43, 44). As far as myocarditis is concerned, it is believed to involve three phases: the acute phase of innate immune activation, the subacute phase of adaptive immune activation, and the chronic inflammatory phase (40, 45). Activated innate immune cells and cardiac cells release cytokines, chemokines, interferons, and alarmins, leading to further activation and recruitment of innate immune cells to the heart, including mast cells, neutrophils, dendritic cells, monocytes, and macrophages (3, 40). Antigen presentation activates adaptive immunity, especially T-lymphocyte subsets, and excessive or persistent activation is an essential factor that can cause and aggravate chronic inflammation. Inflammation, necrosis, and remodeling can lead to cardiac dysfunction (40). Pericarditis may be accompanied by myocarditis or CAI and pleural effusion in patients with EBV. According to the available literature, although EBV is a rare but recognized cause of pericarditis, which can lead to pericardial effusion and even cardiac tamponade, it was detected in the pericardial effusion in only a few patients (14, 15). In addition, valvular heart disease associated with EBV, which may be primary or secondary, is mainly considered to be induced by EBV-associated vasculitis despite limited evidence in patients with CAEBV or EBV-HLH (8, 21, 23, 31). Heart failure is a symptom and sign of cardiac dysfunction. Undoubtedly, all EBV-related organic diseases of the heart, including myocarditis, CAI, pericardial effusion, and cardiac valvulopathies, could lead to heart failure (14, 17, 31, 32).

## 3 Different cardiovascular manifestations and diagnosis in patients with EBV infection

The databases Pubmed, Embase, Web of Science, Sino-Med, Cochrane, CNKI, VIP, and Wangfang Data were searched to gather data on cardiovascular damage brought on by EBV infection from inception to November 2022. There were 28 articles (with a total of 54 patients included) reporting EBV-associated cardiovascular damage: 17 from China (7, 8, 13, 19, 21–31, 34, 35), one from Morocco (12), two from Italy (6, 17), one from Australia (13), three from America (14, 18, 33), one from England (15), and three from Japan (9, 16, 20). There were 10 (18.5%) adults (6, 12–20) and 44 (71.5%) children (under 18 years old) (7–9, 21–35), ranging in age from 1 year and 5 months to 67 years. In terms of sex, there were 28 male (7, 12, 16, 18, 20, 23, 25, 27, 28, 30–34) and 26 female patients (6, 8, 9, 13, 15, 17, 19, 21, 22, 24, 26–29, 31, 35). Regarding the diagnosis of EBV infection, 23 (42.6%) were diagnosed with AEBV (6, 13–15, 17, 18, 25, 27, 32), 20 (37.0%) with CAEBV (7, 8, 16, 19–22, 28–31, 34), 6 (11.1%) had a complication of CAEBV and HLH (12, 24, 28, 31), 4 (7.4%) had a diagnosis of EBV-HLH (9, 23, 33, 35), and 1 (1.9%) had reactivated EBV (26).

**Figure 2:**
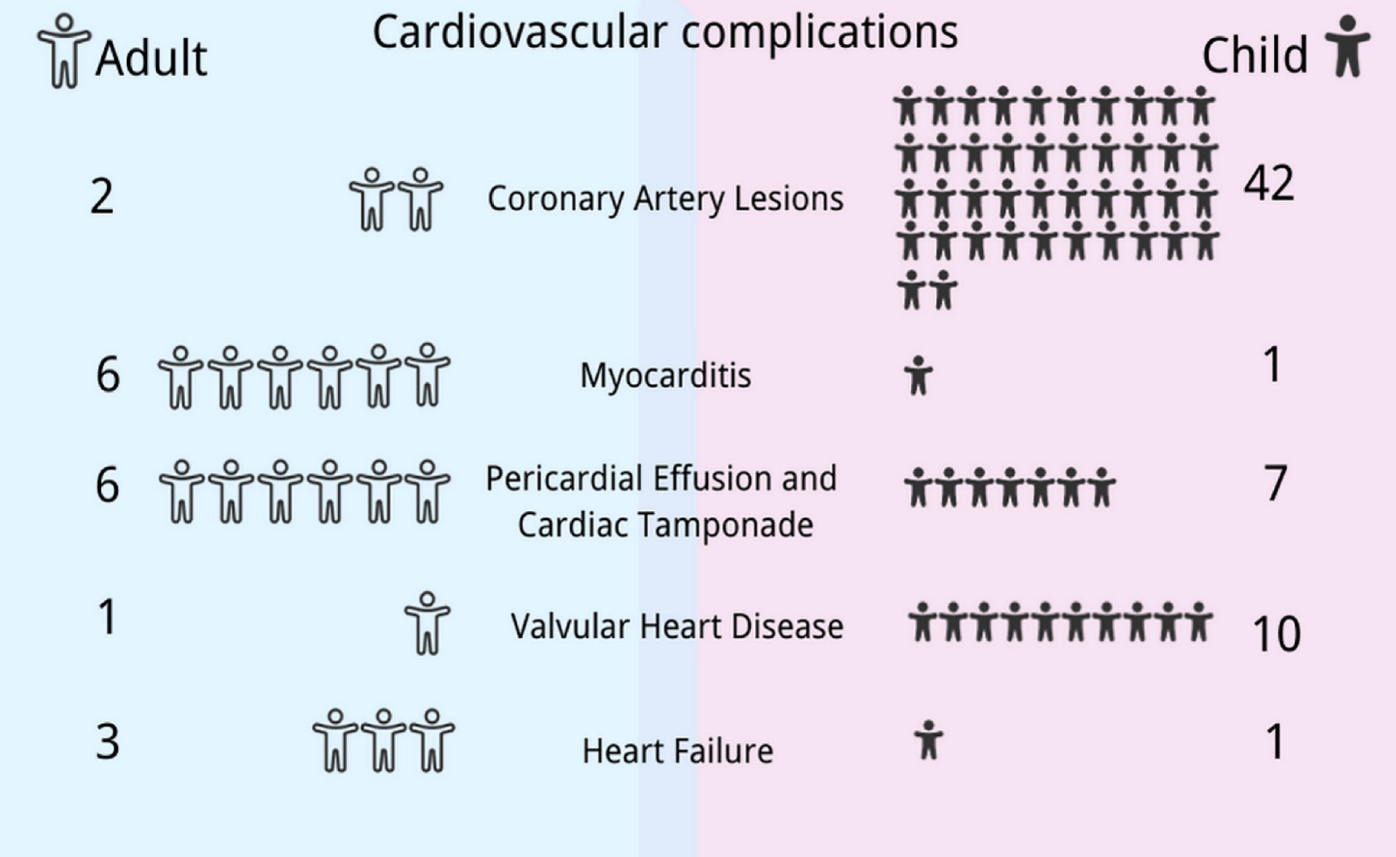
Differences in cardiovascular manifestations in Epstein–Barr virus (EBV) infection between children and adults. The spectrum of cardiovascular manifestations associated with EBV differs between children and adults: coronary artery lesions, valvulopathy, and pericardial effusion are more common in children, while myocarditis and heart failure usually occur in adults. This figure was drawn with the Canva software and the health icons accessed at https://healthicons.org/.

We found that most of the reported cases of EBV-associated cardiovascular complications occurred in China and Japan, with roughly equal gender ratios. Among the cardiovascular complications related to EBV infection, CAA, CAD, valvulopathy, and pericardial effusion are more common in children, while myocarditis and heart failure usually occur in adults (**Figure 2**). Details are classified in the following sections.

### 3.1 Coronary artery lesions

Coronary artery lesions (CALs), also called CAI, are determined based on the measurement of luminal dimensions using echocardiography or coronary angiography. Instead of absolute coronary artery dimensions, the *Z* score (diameter assessment based on body surface area) has become more widely used for CAI in recent years, which is classified into five categories: no involvement (*Z* score <2), only CAD (*Z* score 2–2.5), small CAA (*Z* score ≥2.5 to <5), medium CAA (*Z* score ≥5 to <10 and absolute dimension <8 mm), and large and giant CAA (*Z* score ≥ 10 or absolute dimension ≥8 mm) (46). Patients with CAI can present with various manifestations, such as chest discomfort, congestive heart failure, acute coronary syndrome, and sudden cardiac death, or they may be entirely asymptomatic (47). Coronary artery abnormalities in adults are most frequently caused by atherosclerosis, and EBV infection is a significant cause of CAA (48). CALs in children include congenital and acquired causes, and the most common acquired factor is Kawasaki disease (KD), followed by EBV infection. In the past, less attention had been paid to CAI associated with EBV, but it seems not to be a rare cause.

It is not difficult to diagnose coronary artery abnormalities, whereas it is challenging to determine whether CALs refer to EBV infection or KD, particularly incomplete KD. Patients under 6 months of age with an inexplicable fever lasting 7 days should be suspected of having an incomplete KD, as should all age groups with an unexplained fever lasting 5 days but meeting only two or three clinical KD criteria (46). In addition, a significant proportion of patients with KD test positive for EBV-DNA PCR, which makes it challenging to distinguish between KD and EBV infection in those with CALs. For suspected KD with erratic symptoms, EBV is thought to be a potential alternative diagnosis (46). The cause of coronary injury cannot be fully determined unless coronary artery tissue is available for EBV DNA testing, which might not be realistic. When histopathology is unavailable, the diagnosis is based on the clinical symptoms and follow-up. In general, neutrophil activation in KD is crucial, while lymphocyte predominance in EBV may help to distinguish cases (49–52). To prevent the progression of CALs, intravenous immunoglobulin (IVIG) will be administered, with a further diagnosis based on the clinical manifestations that remain after treatment.

We retrieved 18 articles (with 44 included patients) reporting EBV-associated CAI, including 15 from China (7, 8, 21–31, 34, 35), two from Japan (9, 16), and one from Morocco (12). Of the included patients, 42 (95.5%) were children (7–9, 21–31, 34, 35) and two (4.5%) were adults (12, 16). Regarding gender, 23 (52.3%) were male (7, 8, 23, 25, 27, 28, 30, 31, 34) and 21 (47.7%) were female (9, 12, 16, 21, 22, 24, 26–29, 31, 35). Pediatric patients ranged in age from 1 year and 5 months to 16 years. Regarding the diagnosis, 16 (36.4%) were diagnosed with AEBV (25, 27), one (2.3%) with reactivated EBV but not CAEBV (26), 18 (40.9%) with CAEBV (7, 8, 16, 21, 22, 28–31, 34), six (13.6%) with CAEBV and HLH (12, 24, 28, 31), and three (6.8%) with EBV-HLH (9, 23, 35).

Of the cases with CAI, 31 (70.4%) had only CAD (23–27, 29, 34), eight (18.2%) had only CAA (8, 9, 12, 21, 22, 28, 31, 35), and five (11.4%) had both CAD and CAA involving different coronary arteries (7, 16, 28, 30), which is a reminder of the possibility of multiple involvements. Among all the patients, there were two giant CAAs (9, 12) and one sinus of valsalva aneurysm (SOVA).

### 3.2 Myocarditis

The symptoms of myocarditis vary from compensation to decompensation, for example, moderate dyspnea or chest discomfort that goes away on its own, cardiogenic shock, and death (53). It is challenging to diagnose and exclude clinically. A definitive diagnosis of myocarditis associated with EBV can be confirmed only by an invasive endomyocardial biopsy and by identifying the viral genome using PCR techniques (54). Even so, the American Heart Association recommends less invasive methods to exclude the most prevalent causes of heart illness, including blood tests for cardiac biomarkers, electrocardiograms, echocardiography, angiography, and cardiac MRI, before pursuing more invasive workups. Of the seven reported patients with EBV-associated myocarditis, only two (28.6%) underwent endomyocardial biopsy (6, 20).

In patients with myocarditis associated with EBV infection, troponin (I/T), creatinine kinase, C-reactive protein, and B-type protein, among others, may be elevated, but blood work is nonspecific. Regarding electrocardiograms, there could be ST segment changes and bundle branch block, both of which indicate myocardial damage, although their frequency varies significantly with the lack of specificity. Echocardiographic findings in patients with myocarditis are nonspecific and varied but may help rule out the valvular cause of heart failure (55). Angiography is often used to identify myocarditis and ACS because the negative findings of angiography help rule out ACS. Cardiovascular MRI has become a reliable, noninvasive method for identifying the inflammatory, edematous, and necrotic symptoms of acute myocarditis during the past few decades.

There were seven articles reporting EBV-associated myocarditis (6, 9, 14, 17, 18, 20, 32), of which two were American (14, 18), two were Italian (6, 17), one was Chinese (32), and two were Japanese (9, 20), with a total of seven patients, consisting of one (14.3%) child (9) and six (85.7%) adults (6, 14, 17, 18, 20, 32). There were four (57.1%) male (14, 18, 20, 32) and three (42.9%) female patients (6, 9, 17). The average age of the adults was 32.5 years (range, 19.25–64 years). Regarding the diagnosis of EBV, five (71.4%) had a diagnosis of AEBV (6, 14, 17, 18, 32), one (14.3%) had a diagnosis of CAEBV (20), and one (14.3%) had a diagnosis of EBV-HLH (9).

### 3.3 Pericardial effusion and cardiac tamponade

Pericardial effusion and cardiac tamponade are pericardial diseases that may be either an isolated disease or part of a systemic condition. Pericardial disease has many potential causes that vary by population and geography. Although pericardial disease is often idiopathic in developed countries, infection, especially viral infection, is also a cause of this disease that needs to be considered (56, 57). The most common pathogens of pericardial diseases are coxsackieviruses and echoviruses; in contrast, EBV is seldom detected. According to the available literature, EBV is a rare but recognized cause of pericarditis that can cause pericardial effusion or even cardiac tamponade, depending on the volume. In terms of the diagnosis of EBV infection, four (30.8%) were diagnosed with AEBV (6, 13, 15, 17), five (38.5%) had a diagnosis of CAEBV (19, 20, 31), one (7.7%) had a diagnosis of CAEBV and HLH (31), and three (23.0%) had a diagnosis of EBV-HLH (9, 23, 35).

Chest X-rays are commonly performed for patients with pericardial effusions because they typically complain of chest discomfort and dyspnea. Chest X-rays often show no abnormalities, but in cases of massive effusion, the heart may appear spherical with a flask-like appearance. Therefore, echocardiography, which has almost 100% accuracy, is advised for all suspected pericardial patients with effusions (58, 59). The majority of individuals with a straightforward pericardial effusion may be diagnosed and monitored by echocardiography. Further CT or MRI imaging is rarely necessary. CT may be an option to consider in some cases. Sometimes, the extent of a pericardial effusion may be determined using CT. Secondly, depending on the level of CT attenuation, CT may reveal information about the makeup of pericardial effusion.

We found 10 articles reporting EBV-associated pericardial effusion and/or cardiac tamponade (6, 13, 15, 17, 19, 20, 23, 24, 31, 35), five of which were Chinese (19, 23, 24, 31, 35), two were Italian (6, 17), one was Australian (13), one was Japanese (20), and one was English (15), featuring a total of 13 patients. Of these patients, seven (53.8%) were children (23, 24, 31, 35), six (46.2%) were adults (6, 13, 15, 17, 19, 20), four (30.8%) were male (20, 23, 31), and nine (69.2%) were female (6, 13, 15, 17, 19, 24, 31, 35). The average age of the children was 4.3 years (range, 1.8–12.2 years), while that of the adults was 51.0 years (range, 32.0–70.3 years).

### 3.4 Valvular heart disease

The heart consists of four valves: two atrioventricular valves, namely, mitral and tricuspid, and two semilunar valves, namely, aortic and pulmonic. These valves play a pivotal role in facilitating circulation through the pulmonary and systemic circuits of the heart. Echocardiography is the gold standard in the diagnosis of valvular heart disease. Valvulopathy caused by EBV infection mainly included regurgitation and insufficiency involving the mitral valve, tricuspid valve, and aortic valve. There is no report in the literature involving the pulmonary valve related to valvulopathy.

There were seven articles reporting EBV-associated heart valve disease (8, 14, 21, 23, 28, 29, 31), six of which were Chinese (8, 21, 23, 28, 29, 31) and one was American (14). Of the 11 patients, 10 (90.9%) were children, with an average age of 6.3 years (range, 4.0–10.9 years) (8, 21, 23, 28, 29, 31), and one (9.1%) was a 20-year-old adult (14). There were five (45.5%) male (14, 23, 31) and six (54.5%) female patients (8, 21, 28, 29, 31). Regarding the diagnosis of EBV infection, one (9.1%) was diagnosed with AEBV (14), eight (72.7%) with CAEBV (8, 21, 28, 29, 31), one (9.1%) with both CAEBV and HLH (31), and one (9.1%) with EBV-HLH (23).

Valvular heart disease associated with EBV infection may be primary or secondary. A 20-year-old male patient with myocarditis, dilated cardiomyopathy (DCM), and moderate-to-severe mitral and tricuspid regurgitation was reported (14). Another two papers reported two 4-year-old female children diagnosed with CAEBV with coronary aneurysm, left ventricular hypertrophy, and mitral valve and aortic insufficiency (8, 28). In the three aforementioned cases, DCM and left ventricular hypertrophy were present (8, 14, 28). Although it is difficult to establish a causal relationship between them and valvular heart disease, the possibility of valvular regurgitation and left ventricular hypertrophy, secondary to DCM, cannot be ruled out, and five studies reported aortic regurgitation (AR) in CAEBV or EBV-HLH.

### 3.5 Heart failure

Heart failure is a condition with signs and symptoms brought on by heart malfunction, which shortens life expectancy (60). Heart failure related to EBV infection has only been reported in four studies with four patients who had heart failure caused by myocarditis associated with EBV infection (14, 17, 20, 32). Of the four patients, one was a child (32), three were adults (14, 17, 20), three (75.0%) were male (14, 20, 32), and one (25.0%) was female (17). The average age was 20 years (range, 17.8–38.8 years). As for the diagnosis of EBV infection, three (75.0%) had a diagnosis of AEBV (14, 17, 32) and one (25.0%) had a diagnosis of CAEBV (20).

## 4 Treatment

The treatment and management of cardiovascular involvement associated with EBV infection remain challenging. According to the existing literature, CALs are more common in CAEBV and EBV-HLH, while myocarditis occurs more often in AEBV. As mentioned previously, EBV-related cardiovascular involvement is classified into direct and immune injury. Consequently, its treatment mainly includes three parts: antiviral therapy, anti-inflammatory therapy, and the management of cardiovascular complications.

Antiviral therapy, such as acyclovir and ganciclovir, can decrease viral replication during the acute phase, but with uncertain efficacy in patients with CAEBV and EBV-HLH. Inflammation can be reduced or blocked by glucocorticoids and other immunosuppressants, but complete remission of CAEBV and EBV-HLH requires drug chemotherapy or hematopoietic stem cell transplantation (SCT).

As for specific cardiovascular complications, different manifestations should be managed according to relevant guidelines. The treatment of CALs mainly involves antiplatelet therapy, anticoagulant therapy, and medications that inhibiting or reverse remodeling. If there is thrombosis and/or embolism, long-term use of antiplatelet and anticoagulant drugs should be considered (61). Aspirin is usually the go-to anticoagulant for medical therapy, and clopidogrel is an alternative or combination. Patients with large CAA often require a combination of antiplatelet and anticoagulant drugs in order to prevent thrombotic events (46). Clinical studies have not been able to support the use of angiotensin-converting enzyme inhibitor (ACEI) and angiotensin receptor blocker (ARB) medications as standard treatments for EBV-associated CAI. In fact, there is no evidence suggesting potential benefits to those who receive these medications. When dealing with acute myocarditis, it is important to closely observe cardiovascular health and heart rhythm and provide the necessary support, similar to the approach taken with cases of acute heart failure (62). If there is low cardiac output, immediate steps, such as administering inotropic medications and vasopressors, must be taken to rectify the condition (62). Furthermore, if a dangerous irregular heart rhythm arises, implanting a temporary pacemaker should be taken into consideration as soon as possible (62).

As for pericardial effusion and cardiac tamponade, the evaluation should be conducted using echocardiography, and the treatment strategy is straightforward, with pericardiocentesis being the mainstay, depending on the volume and hemodynamic stability (63).

Valvular heart disease should be stratified according to risk assessment for comprehensive treatment, and severe patients should be treated with surgical intervention as soon as possible (64). However, in the reported cases, none of the patients with valvulopathy received this procedure.

## 5 Prognosis of different cardiovascular manifestations

### 5.1 Coronary artery lesions

When it comes to the prognosis of coronary artery abnormalities, the outcomes of CAA are more pessimistic regarding the risk of thromboembolism and aneurysm rupture compared to CAD. More than half of cases with CAD can return to normal, but CAAs often remain stable and require long-term medication. The prognosis for CALs is also related to the type of EBV infection. According to the reported literature, all patients with CAI progression are CAEBV patients.

Among the reported cases of CAD associated with EBV, CAD returned to normal in 19 (52.8%) patients (23, 25–27, 31), and the required period ranged from 2 to 124 days. CAD did not recover in 11 (30.6%) patients (27–29, 31), while it progressed in four (11.1%) patients (16, 24, 28, 34). The follow-up of CAD remained unclear for two (5.6%) patients (7, 30). Regarding the prognosis, two patients eventually died (24, 31), one of an unknown cause due to giving up treatment after being diagnosed with CAEBV (24) and the other of a severe infection related to hematopoietic SCT (HSCT) (31). The rest of the patients were alive.

There were 12 patients with CAA, two (16.7%) of whom died (12, 21) and one (7.7%) progressed (16), while the majority of these patients remained stable and needed to be kept on medication. The two patients who died suffered from giant CAA, eventually dying of aneurysm rupture (12) and of an unknown cause after giving up treatment (21), respectively.

### 5.2 Myocarditis

The prognosis for patients diagnosed with myocarditis likely depends on multiple factors, including the severity of the presentation, location, expertise of care, and the timing of potential treatment. Myocarditis has a poorer prognosis if it progresses to DCM or if malignant arrhythmias or heart failure is present. Among the seven patients (6, 9, 14, 17, 18, 20, 32), four progressed (14, 17, 18, 32): one had heart failure (14), one experienced cardiopulmonary arrest (18), one presented heart failure and arrhythmias (32), and one suffered from arrest and arrhythmias (17). Three (43%) patients died (14, 18, 20), one of arrhythmia (14), one of multiple embolic infarcts (18), and one of multiple organ failure and disseminated intravascular coagulation (DIC) (20). In summary, one patient died of cardiac causes, while the rest died of multiple organ damage caused by EBV infection.

### 5.3 Pericardial effusion and cardiac tamponade

In the retrieved literature, most of the pericardial effusions caused by EBV were small amounts of fluid, and no special treatment was performed. Two patients presented with severe pericardial effusion coupled with subacute cardiac tamponade (15, 30), while two others presented with a massive pericardial effusion (19, 35). These patients received pericardiocentesis (15, 19, 30, 35).

Pericardial effusion caused by EBV infection does not lead to a poor prognosis unless combined with other cardiac complications. Furthermore, it was found that the pericardial effusion in most patients completely disappeared during follow-up.

### 5.4 Valvular heart disease

Valvular regurgitation associated with an EBV infection can be asymptomatic or can manifest as heart failure, depending on the degree of valvular disease. The prognosis of heart valve disease caused by EBV infection is considered good. Among 11 patients with EBV-associated valvular heart disease, three (27.3%) died (14, 21, 28): one patient had moderate-to-severe heart valve regurgitation and eventually died of heart failure (14), one gave up treatment and died of unknown causes (21), and another died from mixed infection (28). The rest of the participants had mild to moderate valvular disease and were in a stable condition.

### 5.5 Heart failure

In one case, myocarditis progressed to DCM and eventually to congestive heart failure (14). In the other three cases, fulminant myocarditis led directly to acute heart failure. The prognosis of heart failure is poor. Of the four patients reported, two (50%) died (14, 20) and two (50%) were alive (17, 32).

Briefly, among the various cardiovascular damages mentioned above, the prognosis for CAD and pericardial effusion is relatively good. In more than half of the patients, CAD returned to normal, and pericardial effusion disappeared in all patients. The prognosis of other cardiovascular complications is related to EBV infection, the severity of cardiovascular damage, and the involvement of other organs, and the mortality rate is approximately 15%–50% when calculated with the current data in **Table 1**. Undoubtedly, clinicians should pay more attention to the cardiovascular involvement associated with EBV, and cardiovascular evaluations and the management of patients with EBV are essential. Future research on biomarkers is needed in order to identify patients at high risk of cardiovascular complications related to EBV.

## 6 Conclusion

EBV infection is a significant contributor to acquired cardiovascular damage with various manifestations, including CAL, myocarditis, pericardial effusion, cardiac tamponade, valvular heart disease, and heart failure, among others, which presents challenges to the clinical diagnosis and treatment and should be paid attention to. Evaluation of cardiovascular complications in individuals with EBV is necessary for timely intervention and long-term management. The prognosis is not always optimistic, and more research is still needed in the future to promote in-depth understanding.

## Author contributions

XC, YL, LD, JY, BH, and XX conceived and conceptualized the manuscript. XC, YL and LD drafted the manuscript. YL and XC contributed to the figure. LW, WZ, JH, and LC made contributions to the table and data summary. XC, YL, and XX critically reviewed and revised the manuscript for important intellectual content. All authors contributed to the article and approved the submitted version.
